# Porous Alginate Scaffolds Assembled Using Vaterite CaCO_3_ Crystals

**DOI:** 10.3390/mi10060357

**Published:** 2019-05-29

**Authors:** Alena Sergeeva, Anna S. Vikulina, Dmitry Volodkin

**Affiliations:** 1 Fraunhofer Institute for Cell Therapy and Immunology, Branch Bioanalytics and Bioprocesses, Am Mühlenberg 13, 14476 Potsdam-Golm, Germany; alenasergeeva@mail.ru; 2 School of Science and Technology, Nottingham Trent University, Clifton Lane, Nottingham NG11 8NS, UK; dmitry.volodkin@ntu.ac.uk

**Keywords:** calcium alginate, porous hydrogel, polymer scaffold, calcium carbonate, encapsulation, drug delivery, cell culture

## Abstract

Formulation of multifunctional biopolymer-based scaffolds is one of the major focuses in modern tissue engineering and regenerative medicine. Besides proper mechanical/chemical properties, an ideal scaffold should: (i) possess a well-tuned porous internal structure for cell seeding/growth and (ii) host bioactive molecules to be protected against biodegradation and presented to cells when required. Alginate hydrogels were extensively developed to serve as scaffolds, and recent advances in the hydrogel formulation demonstrate their applicability as “ideal” soft scaffolds. This review focuses on advanced porous alginate scaffolds (PAS) fabricated using hard templating on vaterite CaCO_3_ crystals. These novel tailor-made soft structures can be prepared at physiologically relevant conditions offering a high level of control over their internal structure and high performance for loading/release of bioactive macromolecules. The novel approach to assemble PAS is compared with traditional methods used for fabrication of porous alginate hydrogels. Finally, future perspectives and applications of PAS for advanced cell culture, tissue engineering, and drug testing are discussed.

## 1. Introduction

At present, in the field of biomedical technologies, researchers have been attracted to the development of novel multifunctional structures with structure and properties well-controlled on both the micro- and nano-scales. One of the major focuses in tissue engineering and regenerative medicine is the formation of polymer scaffolds, temporal or permanent constructions providing both mechanical support for cell seeding and growth, as well as encapsulation/protection and controlled delivery of bioactive molecules (for instance, growth factors and enzymes) in order to guide tissue organization. Besides the tissue engineering for regenerative medicine, such scaffolds can serve as a platform for animal-free drug testing. Up to date, fabrication of multifunctional scaffolds that have a well-defined structure remains challengeable due to a high level of complexity in the composition of such scaffolds and the need to employ sophisticated methods for the scaffold assembly. In addition, conditions of scaffold preparation often require intolerably high costs of exclusive techniques and often result in a loss of bioactivity of bioactives loaded into scaffolds. Thus arises the need to develop simple strategies for the manufacture of *intelligent* polymer-based scaffolds possessing a well-controlled internal structure, efficient encapsulation, protection, and controlled release of desired bioactives (recent reviews [1,2,3,4]).

**Figure 1:**
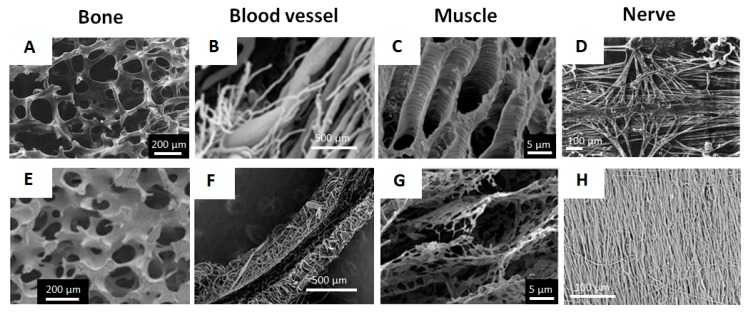
SEM/cryo-SEM images of mammalian tissues (**a**–**d**) and porous polymer-based scaffolds that mimic corresponding tissues (**e**–**h**): (**a**,**e**) human trabecular bone. Reproduced with permission from [5], published by Springer Nature, 2011 and [6], published by Elsevier B.V., 2012; (**b**,**f**) human uterine peripheral vessels. Reproduced with permission from [7], published by Via Medica, 2004 and [8], published by Elsevier B.V., 2005; (**c**,**g**) porcine muscular tissue. Reproduced with permission from [9], published by Springer Nature, 2018; (**d**,**h**) human transverse cervical nerves. Reproduced with permission from [10], published by Springer-Verlag, 1977 and [11], published by Springer Nature, 2019

Polymeric 3D scaffolds serve as the supports to guide the growth of biological cells and the development of a microtissue; often these scaffolds are biodegradable. Polymeric scaffolds are usually designed as porous structures with highly developed internal surfaces to ensure cell infiltration/growth and to avoid diffusion limitations for transport of nutrition and metabolites. This also mimics the architecture of natural tissues well (Figure 1).

Among the others, alginate hydrogels are one of the pivotal materials used for the fabrication of polymeric scaffolds due to alginate biocompatibility, opportunity to shape alginate hydrogels into a variety of sophisticated geometries and topologies. This is possible in both 2D (e.g., thin films patterned with microwells [12] and gel grids [13]) and in 3D (sponge-like structures [14] and gels possessing tube-like [15] or spherical pores [16,17]).

In recent years, a novel bench-top method has been proposed for the fabrication of porous alginate scaffolds (PAS) [18,19]. These scaffolds are produced by formulation of alginate mixture with vaterite calcium carbonate microcrystals (cores), followed by elimination of the CaCO_3_ cores under mild conditions including physiological pH. This is accompanied by the release of Ca^2+^ ions inducing the cross-linking of the alginate gel and formation of hollow pores as inverse replica of the cores. Schematic representation of the process of PAS formation using calcium carbonate is given and discussed in details in Section 4.1 of this review. PAS have highly developed internal structure and offer unique opportunities to host bioactive molecules of a different nature via proper localization of them in the scaffold and to release these molecules in a controlled manner [18,19].

This review summarizes different aspects of the PAS formation discussing current achievements and challenges in this field. Critical comparison of PAS with other approaches proposed to tackle the problems associated with the design of multifunctional scaffolds allows for manifesting high potential of the novel developed technology for tissue engineering, regenerative medicine, drug testing and other applications where multifunctional polymer-based scaffolds are currently employed and strongly required.

## 2. Ca^2+^-Alginate Gel Based Scaffolds

### 2.1. Chemistry of Alginate

**Figure 2:**
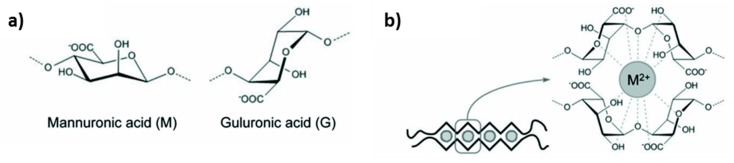
(**a**) Chemical structure of α-L-guluronic and β-D-mannuronic acids (residues of alginate macromolecules). (**b**) Schematics of the binding between G-blocks of alginate molecules by means of divalent metal ions (M^2+^).

Alginic acid is a naturally-derived block copolymer forming polyanions which macromolecule is composed of α-L-glucoronate and β-D-mannuronate units (Figure 2a) [20,21,22,23,24,25]. Sequence, and hence the properties of alginic acid, depend on the natural source. Commercially available alginic acid is produced from brown algae and usually available as in the form of the salt called alginate; molecular weights of these alginates typically vary from 32 to 400 kDa [22]. Since dissociation constants (*pK_a_*) of carboxylic groups of alginate are 3.65 and 3.38 for α-L-glucoronate and β-D-mannuronate residues, respectively, [21,26] in order to dissolve alginate, it is essential to achieve pH above a certain critical value, higher than the *pK_a_*. Besides this, viscosity of alginate depends on the ionic strength and, remarkably, the addition of some ions causes alginate gelation. The latest provides great advantages to alginate compared with the other polysaccharides (gelatine, agar) because alginate is able to form a gel in the range 0−100 °C. Moreover, alginate gels are highly hydrated, having water content > 95%, and can be heated without melting (phase transition) [24].

Chemical modification of alginate is widely employed to provide the polymer with novel desired properties (solubility, hydrophobicity, affinity to specific molecules, etc.) [20,22]. Thus, the phosphorylation of alginate results in the enhancement of hydroxyapatite nucleation and growth. Alginate sulfonation has been applied to provoke anticoagulant activity of the alginate. The attempts to transform hydrophilic alginate to a molecule with hydrophobic or amphiphilic properties were also demonstrated. The other way to provide alginate with new properties is based on the graft copolymerization. Alginates can also be functionalized with specific cell-targeting ligands in order to strengthen the affinity of alginate gels to biological cells. In the next section, the gelation and properties of alginate hydrogels will be discussed.

### 2.2. Alginate Hydrogels: Formation and Structure

Alginate hydrogels are highly hydrated 3D cross-linked polymer networks [20,22,24]. In general, alginate molecules chelate with multivalent cations. This process leads to the gelation occurring via the precipitation of alginate-cation complex and hence the formation of ionically cross-linked gels, also widely called physical gels. The chemistry behind this process is based on the cooperative binding between glucoronates (G-blocks), between mannuronates (M-blocks) and between glucoronates with mannuronates (MG-blocks). Of note, the binding between G-blocks is the most pronounced, although all types of the binding strongly depend on the type of the gel-forming cation. For instance, divalent ions Ca^2+^, Ba^2+^, and Sr^2+^ bound mainly to GG−dimers, while trivalent lanthanide ions such as La^3+^, Pr^3+^, and Nd^3+^ prone to bind to both GG− and MM−segments. This molecular organisation results in the formation of a diamond-shaped hole consisting of a hydrophilic cavity with the multivalent cation that coordinates oxygen atoms from the carboxyl groups of alginate (Figure 2b) [20,21,22,25,27,28]. The size of the cooperative unit is estimated to consist of more than 20 monomers [25]. It has been demonstrated that alginate affinity to cations increases in the order of Mn < Zn, Ni, Co < Fe < Ca < Sr < Ba < Cd < Cu < Pb [24,29]. This is directly related to the ionic radius and coordination number of cross-linking cations [23] and can be used to tune the properties of the hydrogel. Among others, Ca^2+^ cross-linked alginate gels have an advantage of a high biocompatibility, while the use of other cations may be limited due to the toxicity issue. Ca^2+^ cross-linked alginates gels are predominantly formed via the binding of glucuronic segments. Because of this, the strength of alginate gel is significantly influenced by the content of G-blocks. In general, the higher the gel strength, the lower its elasticity [21]. Hydrogels fabricated from alginate enriched with G-blocks form stiff and brittle gels, while high M content results in the formation of rather soft elastic gels [24].

External and internal gelation, as well as gelation upon cooling, represent three main techniques used to formulate ionic alginate gels [20,22,24]. External gelation which is also called a “diffusion method” is based on direct exposure of alginate into a solution containing cross-linking ions (e.g., CaCl_2_). Ca^2+^ ions diffuse from the continuous phase into alginate droplets cross-linking them and forming gel particles [21]. The main disadvantage of this method is the formation of non-uniform alginate hydrogels due to the establishment of a gradient of Ca^2+^ concentration towards the boundary of the hydrogel where it is in contact with the solution of Ca^2+^ and an extremely high rate of the cross-linking reaction [30,31,32]. To some extent, the problem of non-uniform distribution of the cations in external gelation method can be eliminated using alginates of higher molecular weights or carrying out gelation in the buffer solutions containing phosphate ions that also bind calcium ions and, in this way, compete with alginate.

On the other hand, the internal gelation (so-called “*in situ* gelation”) can be applied to avoid the gel inhomogeneity. For this approach, the source of Ca^2+^ ions (usually particles of low-soluble CaCO_3_ or other salts of Ca^2+^) is distributed within the precursor solution of alginate. Slow dissolution of these particles is generally induced by changing pH (e.g., by addition of self-hydrolysing polymer as D-glucono-δ-lactone, GDL), providing constant flow of crosslinking ions to surrounding alginate molecules. This method results in a uniform ion concentration throughout the gel. As an alternative method, gelation upon cooling [23] is based on consequent dissolution of alginate solution and calcium salt in a hot medium of 90 °C followed by cooling. At the temperature of 90 °C, high thermal energy of alginate chains prevents alignment of the polymers required for gelation (Figure 2b) and irreversibly obstructs cooperative binding of the monomers. Further cooling facilitates the formation of an ordered inter-polymer structure that results in the formation of a homogeneous gel matrix [29]. However, the elevated temperatures used in this approach are unsuitable when using labile and fragile bio-macromolecules (e.g., growth factors).

Hydrogel properties are easily tunable via adjusting alginate composition and gel fabrication approach [33,34,35,36,37,38], as well as the type of crosslinking counterions, the ionic strength, the molecular weight of alginate (reflecting its viscosity), pH and the temperature [39,40,41,42,43,44].

Besides ionically cross-linked (physical) hydrogels, alginate can also form covalently cross-linked (chemical) hydrogels. In general, covalently cross-linked alginate gels have higher mechanical and chemical resistance compared to those of physical hydrogels [20]. Chemical hydrogels possess a high stability in a wide range of pH (between 1 and 13), temperature (from 0 up to 100 °C), and various polar solvents and high ionic strength as well [20]. On the other hand, physical hydrogels are reversible because they are formed due to conformational changes, whereas chemical hydrogels form a permanent structure that is irreversible because of configurational changes occurring during hydrogel formation. Largely, this reversibility of physical hydrogels makes them favourable candidates for a variety of biomedical applications. Other advantages of physical and chemical hydrogels are described elsewhere [45]. Further in this review, physical hydrogels will be considered if not mentioned otherwise.

### 2.3. Alginate Gels as Drug Carriers: Encapsulation and Release

The tailor-made structure and widely tuneable properties of alginate hydrogels, as well as their biocompatibility, make them favourable candidates for versatile biological and medical applications. Thus, alginate hydrogels have been extensively developed as nano- or micro-formulations (in a form of gel particles, beads, or capsules) for controlled drug delivery, as well as materials for wound care and engineering of microtissues [20,21,22,23,24]. The opportunity to encapsulate bioactives into alginate hydrogels under mild conditions, as well as release them in a controlled manner, plays a crucial role for all of these applications and will further be considered more in detail. 

Alginate hydrogels are versatile matrices allowing to encapsulate living cells, macromolecules (proteins, growth factors, enzymes, etc.), therapeutic molecules or functional nanomaterials into the gel network preserving their bioactivity and functions [22,23,25,46,47,48,49]. First achieved almost four decades ago, the encapsulation of islet cells into alginate hydrogel gave rise to their use for cell culture and opened new avenues for tissue engineering [29]. Nearly at the same time, alginate particles have been proposed as containers for encapsulation of molecules [24]. To form alginate beads, at first the alginate solution is usually mixed with the solution of the molecules of interest or the suspension of cells. Then, two scenarios for the preparation of alginate beads are possible. The straightforward way is based on the further exposure of this mixture to the solution of cross-linking ions (employing one of the methods described above) that leads to the formation of the large piece of a hydrogel. Further, smaller alginate beads can be obtained via mechanical breakdown of a bulk gel into the particles of a desired size. However, this approach concedes the opposite way that is usually employed. Namely, the alginate solution mixed with an encapsulated component is immersed into the cross-linking solution drop by drop [21,24]. Depending on the droplet fabrication approach, formed alginate particles range from macro dimensions (> 1 mm) down to nano-beads (< 0.2 µm). Notably, encapsulated components can be either homogeneously distributed over the whole bead volume or concentrated into the centre of a gel bead (e.g., one cell per a single bead) [21,23,24,48,49,50]. Thus, alginate macro-beads (1−2 mm) can be fabricated using the extrusion method when alginate is dripped into the CaCl_2_ bath using a syringe. Modification of this method via employment of an electric field, mechanical vibration, or by using a rotating device results in the formation of microbeads (0.2−1000 μm). A variety of other methods for the fabrication of alginate micro-beads have also been reported and are described elsewhere [21,23,24,48,49,50]. Alginate nano-beads (200 nm and less) are typically produced employing nano-vesicles and emulsion droplets as sacrificial templates. This templating strategy allows for designing not only matrix-type but also hollow structures (nano-capsules) that are formed after elimination of the template. Herein, among others we would like to highlight the use of insoluble vaterite CaCO_3_ crystals as they will be a key for the formulation of PAS. The originality of the use of these crystals arises from its ability to simultaneously serve as sacrificial templates and a source of Ca^2+^. 

A high water content and porous nature of alginate gels (pore sizes in the range 5−200 nm) result in a relatively fast diffusion of biomolecules and drugs within the gel [21,22]. Indeed, the release kinetics directly depend on the gel porosity which can be well-tuned by varying the number of cross-linking cations and its type, composition (source and chemical modification, if applicable) of alginate and the size of alginate beads [21]. As a general rule, smaller pores of 12−16 nm are typical for alginate gels prepared using the diffusion method of gelation, while hydrogels prepared via in situ gelation have larger pores.

Strong electrostatic interaction of alginate matrix with the charged encapsulates also affects the release kinetics. For instance, simultaneous encapsulation of multiple drugs (methotrexate, doxorubicin, and mitoxantrone) has been demonstrated in [51]. It was found that methotrexate that does not interact with alginate rapidly liberates from the hydrogel while covalently bound doxorubicin releases with lower rates via chemical hydrolysis of the cross-linker, and mitoxantrone that is ionically bound to alginate releases only after dissociation of the hydrogel. Mild conditions used during the encapsulation and the gelation minimize protein denaturation and degradation, making alginate an excellent candidate for loading of protein-based bioactives. This stimulated a number of studies aimed at loading/release of a wide range of proteins and nucleic acids [22,25].

It is important to note that mammalian cells have no enzymes to cleave alginate chains which make alginate hydrogels non-degradable in mammals. Therefore, molecular diffusion and erosion of the polymer network are the only two factors that determine the release kinetics of bioactives. While the first scenario of erosion-mediated release is typically observed for prolonged release, the second one (diffusion-mediated release) is usually rather fast and accompanied by a low loading efficiency. The latest results in spontaneous leakage of bioactives from alginate beads. Deceleration of diffusion-mediated release has been reported to be achieved via additional protection of the hydrogel beads using the layer-by-layer (LbL) assembled polymer multilayer shell [21,22]. Due to their chemical structure, alginate gels shrink at a low pH and swell at a neutral pH. At a very high pH or in the presence of EDTA or citric acid as cation chelators the cross-linking ions are released that leads to the dissolution of the gel inducing erosion-mediated release. This phenomenon has been widely used for pH-induced release from alginate hydrogels [21].

### 2.4. Alginate Gels for the Design of Porous Scaffolds

Traditionally, polymer scaffolds for tissue engineering are fabricated using naturally derived biomaterials. Among them, alginate hydrogels have been extensively developed due to their similarity to extracellular matrix of mammalian tissues in terms of mechanical properties and widely tunable kinetics of hydrogel degradation, as well as controlled release of molecules at various pH values including neutral pH [33,52,53]. A wide range of bio-applications of alginate hydrogels includes but not limits to cell transplantation, wound healing, encapsulation and controlled or programmed delivery of drugs and biomacromolecules, and the use as anti-adhesive and repair materials [22,23,32,46,54,55,56,57]. Recent progress in the development of alginate hydrogels for the fabrication of scaffolds showed the employment of a number of advanced techniques including gas foaming [58], 3D printing [59,60], electrospinning [60,61,62], emulsion freeze drying [63], microfluidics [58,64], etc. Alginate gels serve as platforms for cell culture and growth of microtissues [65], cardiovascular muscles [66], bones [67], liver [68,69], etc.

**Figure 3:**
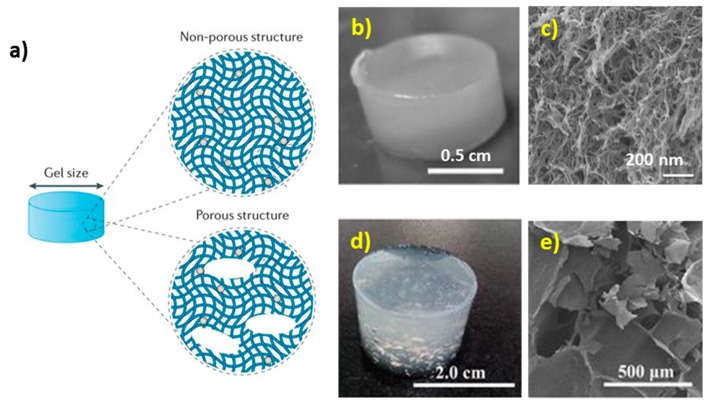
(**a**) Scheme of the architecture of porous and non-porous hydrogels. Reproduced with permission from [70], published by Springer Nature, 2016; (**b**,**d**) Photographs of wet alginate scaffolds and **(c**,**e)** SEM images of dry alginate scaffolds: (**b,c**) — non-porous. Reproduced with permission from [71], published by John Wiley & Sons, 2016; (d,e) — porous alginate scaffold. Reproduced with permission from [72], published by Springer-Verlag Berlin Heidelberg, 2018.

Design of hydrogels on the macroscopic level assumes control over the size and porous structure of the gels [70]. Hydrogel matrices can be either non-porous (having only small pores that are typically in the range of tens of nm for the alginate gel network [71]) or contain macroscopic pores that are typically in the range of 10–500 μm [72] (Figure 3). Dual nano- and macro-porosity is essential for controlled growth of a tissue and drug delivery [70].

High stability of ionically cross-linked alginate gels makes it possible to fabricate a gel with defined dimensions and geometries using different patterning techniques [12,55,73] including light-triggered pattering and employment of microfluidics [54,56,74], electrochemical methods [75], etc. However, utilization of lithography and 3D printing technologies are usually required for design of any hydrogel. The use of harsh conditions during scaffold fabrication (e.g. high or low temperatures, exposure to gas-liquid or solid-liquid interface, the use of organic solvents and aggressive media, surfactants) still remains an obstacle. This often does not allow us to encapsulate bioactives during the hydrogel synthesis that may be crucial for utilization of scaffolds and limits the control over the scaffold internal structure [47].

The latest issue is typically accompanied by scarce pore interconnectivity that is essential for cell colonization in the entire volume of the scaffold. This problem has partially been solved by A. Barbetta et al. [76]. Therein, two methodologies for the formulation of PAS with highly interconnected pores in different size ranges have been proposed. Emulsion templating of the hydrogel allowed producing PAS with the pores of about 10–20 µm that are interconnected via the channels of 2–5 µm [76]. An approach based on the foam templating results in the formation of alginate gels with large 100–300 µm pores and interconnections in the range of 30–80 µm [76]. Both methods allow one to produce PAS with a highly developed internal macro-sized structure that is crucial for cell growth and proliferation due to a need for a free space for cell colonization and requirements of a non-restricted transport of cell metabolites and essential nutrients. However, both approaches [76] lack precise control over the pore distribution that appears random and does not give any options for loading of therapeutics and growth factors, important for cell attachment, growth and proliferation.

This seems to be one of the major challenges for further progress in this field. To the best of our knowledge, only a few works reported the formation of alginate hydrogels with an opportunity to host macromolecules, and no alginate gels possessing both a well-defined internal structure and loading of bioactives at desired doses have been reported. In this sense, one of the most promising methodologies is the templating of alginate hydrogels on mesoporous vaterite CaCO_3_ crystals (the strategy that has been briefly mentioned above). Explored by Wang et al. [16] and by Roberts and co-workers [17], this idea has been implemented employing model molecules (ibuprofen [16] and bovine serum albumin (BSA) [17]) that have been pre-loaded to CaCO_3_ vaterite crystals. A suspension of these crystals has been mixed with the solution of alginate followed by addition of glucono-δ-lactone that slowly doped H^+^ due to its hydrolysis. The acidification caused a mild dissolution of CaCO_3_ and release of Ca^2+^ that cross-link the alginate forming a gel. Macro-sized pores have been formed as a result of calcium carbonate dissolution in the places where CaCO_3_ crystals have been initially located. Consequently, ibuprofen [16] and BSA [17] have been rapidly liberated from the PAS. Additional coating of CaCO_3_ with a polyelectrolyte multilayer shell resulted in slowing down the release rate by ca 50 times as compared with alginate gels formed using bare CaCO_3_ crystals [17]. These works manifested that the utilization of CaCO_3_ crystals as soluble cores for templating alginate hydrogels is a powerful approach promising for the development of scaffolds towards cell-based applications. However, the approach above is rather sophisticated and involves multiple steps including a rather long procedure of multilayer coating bringing additional costs. The way proposed to avoid these issues and novel achievements in CaCO_3_-assistant formation of PAS will be addressed in the Section 4 of this review. However, prior to this, the following Section 3 will describe the structure, principles of the fabrication, and featured properties of vaterite CaCO_3_ crystals.

## 3. CaCO_3_ Vaterite Crystals: Loading and Release Opportunities

### 3.1. Morphology of Vaterite Caco_3_ Crystals

**Figure 4:**
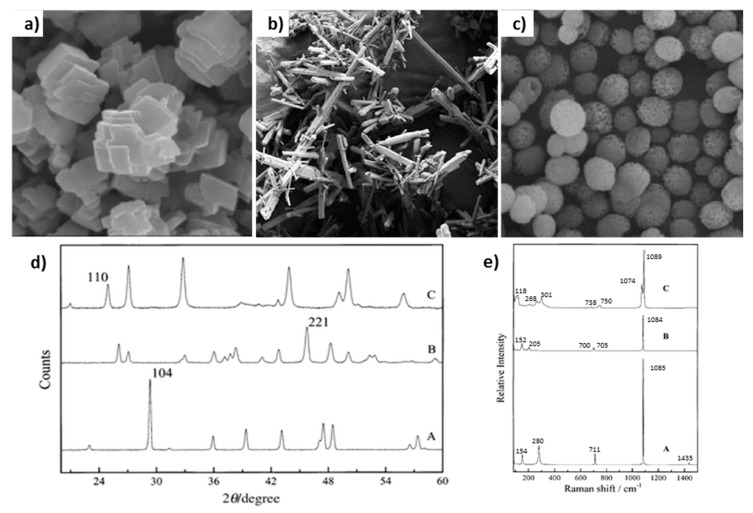
Scanning electron microscopy (SEM) images of CaCO_3_ polymorphs: (**a**) rhombohedral calcilte, (**b**) needle aragonite and (**c**) spherical vaterite. Reproduced with permission from [85], published by Elsevier, 2016. (**d**) XRD and (**e**) Raman spectra of the synthetically prepared calcite (A), aragonite (B) and vaterite (C). Reproduced with permission from [86], published by Royal Society of Chemistry (Great Britain), 2000.

Calcium carbonate mainly exists in the form of one of three polymorphs: calcite, vaterite and aragonite (Figure 4a–c). All the polymorphs have different shapes and morphologies that can be distinguished from each other employing various methods, for instance X-ray diffraction (Figure 4d) or Raman spectroscopy (Figure 4e). Among all of the polymorphs, vaterite is the most attractive for biomedical applications because it has a highly developed internal structure ideal for microencapsulation/release of bio-macromolecules and drugs. Vaterite CaCO_3_ crystals can easily be formed upon mixing of aqueous solutions of precursor salts of Ca^2+^ and carbonate ions. The mechanism of crystal growth is expletively described elsewhere [77,78]. Briefly, spherical vaterite crystals comprise of small nanocrystallines interconnected to each other forming mesoporous structure of the crystal. The use of organic additives [79], some protein/polymer matrices [80] or nanoparticles [81] can direct the growth of vaterite crystals of specific shape and morphology. The porosity of the crystals can also be controlled, e.g. via the variation of crystal preparation temperature [82]. The typical sizes of crystals range from 3 to 20 µm, although a number of recent studies proposed novel ways for the fabrication of nano-crystals [79,80,83] or large vaterite of sizes in sub-millimeter range [84].

### 3.2. Vaterite CaCO_3_ as Decomposable Templates for Microencapsulation

Nowadays, inorganic crystals of the vaterite polymorph of CaCO_3_ are classified as advanced biodegradable and biocompatible materials to be employed for a wide range of bio-applications such as biomedical engineering, biosensors and controlled drug delivery. The growing interest in vaterite CaCO_3_ has emerged based on crystal highly porous nature, easy adjustment of dimensions and porosity during the crystal synthesis, cost-effective formulation and marginal toxicity [87,88,89,90,91]. Indeed, the internal structure of vaterite crystals is mesoporous with the typical pore size of tens of nanometers that is highly favorable for the loading of bio-macromolecules and drugs as well as functional materials such as inorganic nanoparticles (e.g., magnetite [92,93,94,95], silver [95,96]), carbon nanotubes and halloysites [97]. CaCO_3_ crystals can be loaded with the low-molecular-weight molecules, e.g., small drugs (doxorubicin [98]) and photosensitizer [99], as well as with high-molecular-weight macromolecules, e.g., dextrans [88,90], polymers (alginate [90], mucin [100,101]), and proteins (catalase, BSA, insulin [88,89,90,102,103]). The functionalization of CaCO_3_ vaterite crystals with inorganic nanoparticles brings new properties desired for the use of crystals in surface enhanced Raman microscopy [96,104,105,106], making crystals sensitive to external stimuli (e.g., electrical/magnetic fields, light irradiation [107,108,109,110]). The fabrication of pure protein [102,111,112] or polymer [113,114,115] particles can be achieved via hard templating on the vaterite cores. The templating is based on filling the crystal pores with material of interest followed by the crystal elimination that results in the formation of the inverted crystal replica (in case of a full filling of the pores). This opens new avenues for the utilization of vaterite CaCO_3_ crystals and hybrid structures assembled on them as sacrificial templates [116], for controlled release, targeted drug delivery [83], surface patterning [117], and reconstitution of artificial cellular compartments [118].

Impregnation of the encapsulates into the interior of the vaterite CaCO_3_ crystals can be performed at mild conditions in one of two ways: i) during the crystal growth (so-called co-precipitation or co-synthesis) or via the post-loading of the pores of pre-formed crystals (by means of adsorption or via solvent evaporation) [81,89,90,102,119,120]. All methods for the encapsulation have their advantages and disadvantages; the choice of appropriate approach mainly depends on the nature of the encapsulate. Thus, post-loading by means of adsorption represents the mildest method suitable for the encapsulation of fragile macromolecules that are highly sensitive to their microenvironment and can easily lose their bio-activity [120,121]. In its turn, the co-precipitation approach is based on the inclusion of encapsulates into one of the precursor salt solutions to make the crystals, further mixing of the salts and entrapment of encapsulates inside the growing crystals. This leads to higher encapsulation efficiencies if compared to the adsorption method, but may result in a partial loss of the bio-activity of the encapsulated molecules caused by the influence of crystal growth conditions [121]. On the other hand, the co-precipitation provides a homogeneous distribution of molecules within an interconnected internal volume of vaterite crystals. Finally, solvent evaporation grants the highest encapsulation efficiencies, yet it is however the harshest method among three approaches described above due to conditions of solvent removal and it is thus less suitable for labile [121] and sensitive molecules [83].

Besides the integration of molecules of interest inside the crystals, deposition of additional coatings onto the external surface of the crystals can also be achieved, e.g. via the LbL assembly of the polyelectrolytes [88,98,109,122,123,124]. Importantly, the multilayer shells assembled on the crystals are fully permeable for ions and small molecules that allows for a complete decomposition of CaCO_3_ cores when lowering pH or using chelating agents (e.g., EDTA). This results in the formation of completely hollow polyelectrolyte capsules or capsules of a matrix type that contain a polymer matrix inside [125]. The most attractive feature of multilayer capsules assembled on CaCO_3_ cores is a selective permeability of a multilayer shell that can also be functionalized with some stimuli-sensitive materials (e.g., those responsive to pH [126], infra-red light [127,128]), so the encapsulated molecules can be released from the capsule in a controlled manner [129].

### 3.3. Release from Vaterite Caco_3_ Crystal: Dissolution and Recrystallization

If not considering the case of molecular release from functionalized vaterite CaCO_3_ crystals that is mediated by external stimuli, one can distinguish two main mechanisms of the release from bare CaCO_3_ crystals: dissolution- and recrystallization -mediated release. CaCO_3_ can easily be dissolved at a slightly acidic pH [116] or upon the addition of chelating agents, e.g., EDTA or citric acid (corresponding constants of the binding to Ca^2+^ in the CaCl_2_ solution are *K_a_* ~ 2 × 10^8^ M^−1^, and *K_a_* ~ 10^3.5^ M^−1^ at pH 7 for EDTA and citric acid, respectively). While acidic pH is not desirable for sensitive compounds such as proteins or growth factors, dissolution of CaCO_3_ crystals at neutral pH has a crucial importance for bio-applications providing a complete release of the loaded molecules. 

On the other hand, the immersion of mesoporous vaterite crystals into aqueous media results in a phase transition and spontaneous recrystallization of vaterite to thermodynamically more stable but non-porous calcite. If the crystals have been laden with some molecules of interest, the transformation of the vaterite to calcite provokes the liberation of these molecules from the porous interior of vaterite crystals to external medium [130,131].

It is known that vaterite → calcite recrystallization is to a large extent a surface-mediated process [78,132,133,134] and recrystallization kinetics usually exhibits an exponential-like behavior [81]. The recrystallization kinetics can be controlled via the use of additives. For instance, CaCO_3_-Fe_3_O_4_ vaterite microparticles recrystallize significantly faster if compared with pure vaterite crystals [81]. The LbL assembled polyelectrolyte coating of pre-loaded CaCO_3_ crystals can also effectively regulate molecular and ion transport on the crystal-liquid interface allowing us to program the release kinetics [81].

As a short summary of the described above, unique properties of vaterite crystals are in their i) biocompatibility, ii) ability to trap and retain huge amounts of small and large molecules and nanoparticles of various nature, (iii) opportunity to encapsulate bioactives at mild conditions and neutral pH; and iv) wide range of options for programmed and controlled slow/fast release that is either regulated via crystal dissolution and recrystallization (for the bare crystals) or by external stimuli (for functionalized crystals). In recent years, these features stimulated the idea to utilize vaterite calcium carbonate as sacrificial templates for the fabrication of polymer-based alginate scaffolds. In principle, this strategy can provide simultaneous i) cross-linking and adjustment of hydrogel nanoporosity; ii) control over the macroporosity of porous scaffolds and iii) encapsulation and preservation of fragile bioactives in the entire volume of the scaffold. This makes the use of mesoporous CaCO_3_ crystals for the fabrication of PAS a beneficial and superior approach. Latest achievements in this area are discussed below in Section 4.

## 4. Vaterite CaCO_3_-Assistant Porous Alginate Scaffolds (PAS)

### 4.1. Fabrication Strategy

In a majority of works focused on composite CaCO_3_-alginate gel materials, CaCO_3_ crystals are used as a source for mineralization [135] of the scaffolds and/or as a hardening component for the scaffolds utilized in hard tissue engineering (e.g., [136,137]). Therefore, there was no need to eliminate CaCO_3_ crystals in order to form the pores, and even vice versa, the crystals have been kept in the final scaffold architecture. A straightforward approach for the fabrication of composite CaCO_3_-alginate gel materials was first employed nearly one decade ago and has been based on the simultaneous growth of CaCO_3_ crystals and gelation of alginate hydrogel [138,139,140,141,142]. In this design, calcium carbonate crystals grow in the presence of the gel and get entrapped inside this polymer matrix. This strategy showed its promise for the controlled crystallization of CaCO_3_ crystals. However, it has serious limitations. The major one is a lack of control over the internal structure of the scaffold. Although the structure of the growing crystals can be manipulated via the variation of environmental conditions (polymer concentration, gel composition, etc.), the final distribution of crystals and the macrostructure of the scaffold cannot be controlled since the crystallization of CaCO_3_ is a spontaneous and highly sensitive process. In addition, the presence of calcite and sometimes amorphous calcium carbonate has been detected [139]. For some cases, a significant decrease in the size of CaCO_3_ crystals resulted in the formation of nano-CaCO_3_ that found its application in drug delivery but was not suitable for the fabrication of macro-porous scaffolds [141].

**Figure 5:**
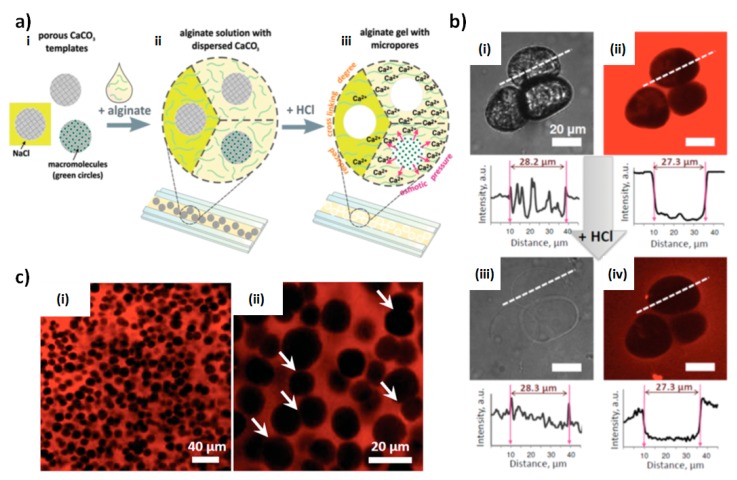
(**a**) Schematics of formation of porous alginate hydrogels. (i,ii) Dispersion of CaCO3 crystals in alginate solution followed by deposition of the suspension onto a glass substrate. (ii,iii) Formation of porous hydrogel by addition of HCl, which induces CaCO3 dissolution. The dissolution process is accompanied by alginate cross-linking and formation of hollow pores. (**b**) Optical transmission and fluorescent images of 33 μm vaterite CaCO_3_ crystals dispersed in alginate before (i,ii) and after (iii,iv) addition of HCl. Fluorescence profiles are given to each image and taken along the white interrupted lines. (**c**) CSLM images of porous alginate gel formed at compact packing of 11 μm CaCO_3_ templates. White arrows in (c,ii) indicate interconnected pores. The gel (b,c) has been stained with rhodamine 6G. Reproduced with permission from [18], published by John Wiley & Sons, 2015.

In contrast, the utilization of vaterite CaCO_3_ crystals as sacrificial cores for the formation of alginate scaffolds in microfluidics set-up allows one to design stable PAS that have a well-defined and highly developed porous structure. The high potential of this fabrication strategy was recently manifested for the precise control over the scaffold porosity [18,19] and a high performance of the encapsulation/controlled release of biomolecules [19]. The work [18] introduced the method offering the fabrication of 2D CaCO_3_-assistant alginate scaffolds at acidic conditions (Figure 5a). Therein, CaCO_3_ crystals suspended in the alginate solution have been spread over a glass substrate (Figure 5ai) followed by the addition of HCl that resulted in the dissolution of calcium carbonate and the release of Ca^2+^ ions that induces physical cross-linking of the hydrogel (Figure 5aii–iii). Control over the concentration of Ca^2+^ and as a result over the cross-linking degree has been achieved via variation of the ionic strength (Figure 5aiii).

### 4.2. PAS Porosity and Mechanical Properties

Gel cross-linking and osmotic pressure generated by the released calcium ions have been shown to play a pivotal role in the formation of the micro-sized pores in PAS [18]. Notably, the pores of the formed PAS are hollow (Figure 5b). The pH used for dissolution of the crystal core is the key to manipulate the stability and size of the pores during CaCO_3_ elimination. Thus, a less acidic pH (that can be achieved by addition of relatively low HCl concentration) results in the slow dissolution of CaCO_3_ that is accompanied by the collapse and closing of the pores. The use of high HCl concentration provokes fast dissolution of vaterite cores that results in the uncontrolled spontaneous formation of CO_2_ bubbles and the enlargement of the formed micro-sized pores. Under optimal acidic conditions, pores keep the size equal to that of the CaCO_3_ crystals used (Figure 5b). This allows a rather easy control over the pore size distribution via the utilization of vaterite crystals of desired dimensions [18].

**Figure 6:**
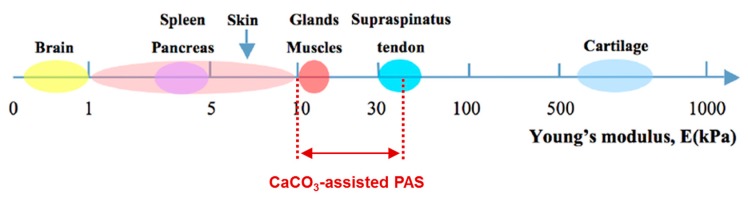
Comparison of Young’s modulus of natural soft tissues and organs and Young’s modulus of CaCO_3_-assisted PASs. Adopted with the permission from [144]. Reproduced with permission from [144], published by MDPI, Basel, 2015.

The structure of the CaCO_3_-assistant PAS assembled on CaCO_3_ crystals of about 11 μm shown in Figure 5c clearly reveals the presence of both closed and interconnected pores. These scaffolds are soft, having the Young modulus of tens of kPa. A highly developed and tunable internal structure and soft nature of CaCO_3_-assistant PAS make them promising for the use in biomedical applications, e.g., for bio-engineering of soft tissues and organs (Figure 6). Another advantage is the opportunity to load and release bioactive molecules into the PAS. Therefore, the last section of this review will highlight recent achievements in the loading/release of non-charged dextrans, charged bio-macromolecules (proteins) and small molecules (dyes) into/out of PAS assembled using vaterite CaCO_3_ crystals.

### 4.3. PAS as Reservoirs for Encapsulation and Controlled Release

**Figure 7:**
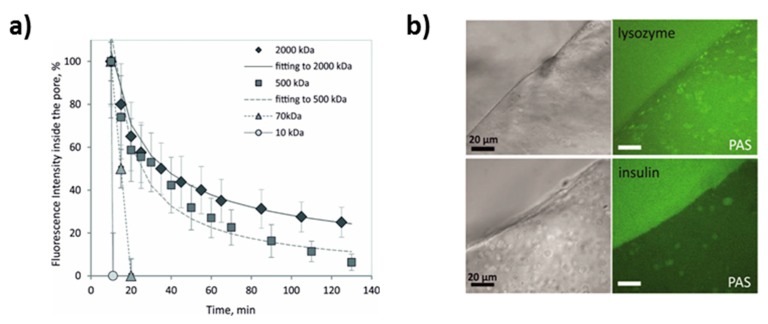
(**a**) Release kinetics of FITC-dextran (different MW) release from the macro-pores of PAS. (**b**) Mass transport of proteins from the solution into/through the PAS gel network. Reproduced with permission from [19], published by American Chemical Society, 2015.

Fluorescein isothiocyanate-labeled dextrans (FITC-dextran) are known as model macromolecules widely employed for investigation of the release performance and kinetics of various carriers. Dextrans have slightly negative zeta-potentials closed to zero at neutral pH and can have molecular weight variable in a wide range [143]. The kinetics of the release of dextrans from vaterite CaCO_3_ crystals has been extensively studied in recent years. The study [19] investigated the release of dextrans of different molecular weights from PASs assembled on ca 8 µm-sized CaCO_3_ cores (Figure 7a). The release rate of FITC-dextran has been demonstrated to be directly related to its molecular weight that is rather typical for homogeneous matrices and indicates a significant role of spontaneous molecular diffusion. Interestingly, alginate concentration had no influence on FITC-dextran release [19]. Assuming the absence of strong electrostatic interaction between dextrans and PAS, it has been concluded that there is a cut off for the molecules of 7–16 nm, so larger macromolecules are retarded by the alginate network and small molecules can freely diffuse the gel outward.

Strong interaction between charged macromolecules (proteins) and alginate gel has also been reported. As opposed to the study of dextran release kinetics, the protein-PAS interaction has been examined assessing the loading of proteins into prepared PAS. Alginate gel itself possesses a negative charge due to a low *pK_a_* of the alginic acid. Small protein lysozyme (oppositely charged compared to alginate gel) accumulates inside PAS (Figure 7b) while negatively charged insulin reaches much lower internal concentration inside the PAS although its diffusion is also rather fast (a scale of minutes) [19]. This clearly indicates a high potential for the encapsulation of macromolecules that possess a positive net charge into the negatively charged PAS. At the same time, the retention of macromolecules processing a negative net charge can be awkward. The scenario described above can, however, be different in some special cases as it has been shown, for instance, for small anionic dye [145].

Therein, the dissolution of phthalocyanine-loaded CaCO_3_ crystals covered by an alginate matrix and re-distribution/release of CuPcTs dye molecules [146] has been monitored by Raman spectroscopy [145]. Notably, small CuPcTs molecules pre-encapsulated into sacrificial CaCO_3_ cores can be retained inside the macro-sized pores formed at the places of eliminated CaCO_3_ crystals that are probably due to the repulsion between negatively charged dye molecules and similarly charged ALG gel surrounding the pores. From the other side, the reason could be the aggregation of small dye molecules inside the pores of CaCO_3_ during the co-synthesis, so such large molecular aggregates cannot escape from the pores due to sterical limitations. In any case, these results are promising for the loading and retention of small and/or for anionic molecules inside the pores of the PAS.

## 5. Summary and Perspectives

Fabrication of porous biopolymer-based scaffolds is rapidly developing field of biomedical engineering. In this field, porous alginate scaffolds built up employing mesoporous vaterite CaCO_3_ microcrystals are extremely promising due to i) highly porous PAS structure that can be well-tuned and ii) the ability to load the scaffolds with bioactive molecules of a diverse nature and release them on demand. Microfluidics-based design of CaCO_3_-assisted PASs utilizing pre-formed CaCO_3_ crystals offers a high degree of control over the internal PAS structure. As opposed to that, the simultaneous crystal growth and alginate gelation lacks the control over the PAS structure and does not provide an opportunity to pre-load CaCO_3_ cores with the desired active compounds.

Despite the high potential of CaCO_3_-assisted PASs, their fabrication and use have not been investigated well yet, and it is better to say that nowadays this approach is only just emerging. Therefore, the design of CaCO_3_-assisted PASs requires a further deep development in terms of fundamental issues raised and applications. Thus, the fabrication of the scaffolds under mild conditions (media with the pH near the physiological one, i.e., pH 7.4) is urgently required as currently employed HCl-mediated leaching of CaCO_3_ cores may result in the reduction bioactives’ activity and cell viability. Thus, the investigation of the interaction of CaCO_3_-assisted PASs with cells will be a crucial step for further development of PASs. There is an intuitive perception that one of the best options would be the substitution of HCl as a relatively aggressive dissolution agent to weaker acids (e.g., citric acid) or chelating Ca^2+^-binding agents (e.g., EDTA). In principle, the latest can be achieved at neutral pH [114].

On the other hand, the strategies used to encapsulate bioactive compounds into CaCO_3_ cores and to protect the scaffolds from undesired spontaneous leakage of these bioactives should further be addressed, verified and improved. Herein, the entrapment of small and/or anionic compounds can turn up the challenge due to the relatively large nano-pores of alginate matrix of the PAS (7–16 nm) and the negative charge of the alginate gel due to carboxylic groups on the alginate backbone. Here, one of the strategies might be the co-loading of these small drugs with large oppositely charged biopolymers. The formation of drug-biopolymer complexes inside the pores of CaCO_3_ promotes the entrapment of the drugs and allows one to substantially increase encapsulation efficiency (e.g., [147,148]). Formation of LbL capsules on CaCO_3_ cores could be alternative strategy.

Pioneering studies on the design of CaCO_3_-assisted PASs indicate that all obstacles mentioned above can potentially be overcome. We believe that the described PASs can become a new generation of biopolymer scaffolds with tailor-made architecture and controlled porosity, high pore interconnection and an opportunity to load and release biomolecules of interest. This allows one to use the terms *intelligent* or *smart* for the PASs, and opens a new avenue for further successful PAS employment towards tissue engineering and regenerative medicine.
